# A Novel Deep Learning-Based Cooperative Communication Channel Model for Wireless Underground Sensor Networks

**DOI:** 10.3390/s22124475

**Published:** 2022-06-13

**Authors:** Kanthavel Radhakrishnan, Dhaya Ramakrishnan, Osamah Ibrahim Khalaf, Mueen Uddin, Chin-Ling Chen, Chih-Ming Wu

**Affiliations:** 1 Department of Computer Engineering, College of Computer Science, King Khalid University, Abha 62529, Saudi Arabia; kanthavel2005@gmail.com; 2 Department of Computer Science, College of Arts and Science-Sarat Abidha, King Khalid University, Abha 62529, Saudi Arabia; dhayavel2005@gmail.com; 3 Nano Renewable Energy Research Center, Al-Nahrain University, Baghdad 10072, Iraq; 4 College of Computing and IT, University of Doha for Science and Technology, Doha 24449, Qatar; mueenmalik9516@gmail.com; 5 School of Information Engineering, Changchun Sci-Tech University, Changchun 130600, China; 6 Department of Computer Science and Information Engineering, Chaoyang University of Technology, Taichung 41349, Taiwan; 7 School of Computer and Information Engineering, Xiamen University of Technology, Xiamen 361024, China; 8 School of Civil Engineering and Architecture, Xiamen University of Technology, Xiamen 361024, China; chihmingwu@xmut.edu.cn

**Keywords:** wireless underground sensor networks, deep learning based cooperative communication channel, multi-input-single-output

## Abstract

Wireless Underground Sensor Networks (WUSNs) have been showing prospective supervising application domains in the underground region of the earth through sensing, computation, and communication. This paper presents a novel Deep Learning (DL)-based Cooperative communication channel model for Wireless Underground Sensor Networks for accurate and reliable monitoring in hostile underground locations. Furthermore, the proposed communication model aims at the effective utilization of cluster-based Cooperative models through the relay nodes. However, by keeping the cost effectiveness, reliability, and user-friendliness of wireless underground sensor networks through inter-cluster Cooperative transmission between two cluster heads, the determination of the overall energy performance is also measured. The energy co-operative channel allocation routing (ECCAR), Energy Hierarchical Optimistic Routing (EHOR), Non-Cooperative, and Dynamic Energy Routing (DER) methods were used to figure out how well the proposed WUSN works. The Quality of Service (QoS) parameters such as transmission time, throughput, packet loss, and efficiency were used in order to evaluate the performance of the proposed WUSNs. From the simulation results, it is apparently seen that the proposed system demonstrates some superiority over other methods in terms of its better energy utilization of 89.71%, Packet Delivery ratio of 78.2%, Average Packet Delay of 82.3%, Average Network overhead of 77.4%, data packet throughput of 83.5% and an average system packet loss of 91%.

## 1. Introduction

Sensor Networks (SNs) are experiencing immense development with the effective utilization of Artificial Intelligence (AI). The incorporation of SNs and AI can make a profit in the business and manufacturing sectors [1,2]. In addition, SNs are extensively used to gather ecological constraints in making appropriate decisions for homes and industrial applications based on the learning experience of day-to-day activities in a real-time manner with AI and Machine Learning (ML). Additionally, with AI, sensor fusion can be carried out more comfortably and precisely than with traditional algorithms [3].

Moreover, neural networks can handle anonymous circumstances in an intelligent way, as they have the ability to become aware of reimbursement methods for the training data and potentially amplify the value of the consequences to the consumer. However, the advancement of AI has the potential to undo the gesture of new sensor applications and push market requirements for smart sensing with the ability to extract information from sensors [4]. Before the original information is transferred using sensors to a system for storing, the original information from sensors must be focused on for collecting the sequence of information. Furthermore, automatically gathered data with tagged data for the preparation of training ML algorithms are integrated; the AI sensors in robots are similar in their provisions such as observation, listening, and manipulation in the same manner as humans [5].

Man-made intelligence in horticulture is supporting ranchers by enhancing their production and reducing indifferent manners. The agricultural trade categorically and straightforwardly uses AI in their training to modify the order. The innovation of AI aids in the control and management of any poor-quality circumstances. Most innovative businesses in horticulture are gradually changing to an AI-empowered manner to tackle the improvement of agrarian formation. AI facilitated mechanisms can recognize environmental changes faster and respond wisely. Organizations in agribusiness, with the assistance of AI, are treating farming data to reduce aggressive outcomes. Simulated intelligence in a high-level manner is assisting the rancher with the information. The required information helps the land-keeper’s high income and profit without being dependent on harvest by understanding and learning AI. In addition, AI is an effective method to recognize potential deformities for DL applications in terms of designs in agribusiness [6].

On the other hand, the Cooperative communication model has been the best choice in complex underground environments for a high density of scalable sensor nodes but without compromising the greater inter-and intra-communication challenges. The cooperative nodes in the sensor network possibly create a well-organized automated dynamic structure to obtain a strong association in signal propagation amid communication hurdles and millimeter waves. When DL techniques and Cooperative modeling are used together in the right way, they can be used to reduce the effects of underground communication limitations such as transmission delay, packet loss, and throughput [7].

The objectives of the paper are two-folded. First, the development of a DL-based Cooperative communication channel model for WUSNs is carried out with the proper utilization of cooperative sensor nodes to reduce the unnecessary energy consumption by individual nodes. Second, the proposed Cooperative communication channel model for WUSNs based on Deep Learning is meant to help people share resources.

The paper is organized into six sections. The literature survey is explained in Section 2. Section 3 elucidates the contributions of DL and ML in the Underground Wireless Sensor Network Environment. The proposed Deep Learning-Based Cooperative Communication Channel Model for WUSNs is explained elaborately. The outcome of the proposed work and experimental results are explained by the DL-Based Transmission Path Selection in WUSN.

## 2. Related Works

In this section, the various DL-based Cooperative communication channel models for WUSNs and comparative analyses are explained through a literature survey.

Zhang et al. [1] provided a survey of DL techniques based WUSNs with possible uses of various communication technologies and frameworks to make computational intelligence implementation on wireless systems effective. They also presented an encyclopedic analysis of DL-based cellular and internet connection research with classification into separate contexts. C. Gungor et al. [8] provided a comprehensive experimental report on the statistical analysis of the wireless medium in various electric power system settings, as well as an underground network converter vault. Additionally, ambient sound, network topology, and amplification in the 2.4 GHz frequency spectrum were also measured for the wireless sensors in real-world power transmission and distribution lines. On the whole, analytical observations and current research provide useful information about IEEE nonionic smart grid platforms to direct selections and note the drawbacks of the Internet of Things (IoT). For solar-powered wireless sensor networks, Ge Yujia et al. [9] suggested a new resource provider focused on cooperative text classification in order to extract electricity more evenly to be distributed across the entire clustered network. In this multi-agent setting, the collective plans of Q-learning and state-action-reward-state-action (SARSA) are being used, with a dependence on parameters such as the node cluster head, projected energy for the subsequent duration slot, and power knowledge of sensor nodes. Their experiments revealed that the proposed method responds well to changing circumstances, improvements in its specifications, and implementation of the quality service specifications. Kisseleff et al. [10] looked at how MI-WUSNs change over time, how signals travel through networks, how core networks work, and how free energy can be converted.

The two main paradigms of the WUSNs providing for estimating signal loss were checked and contrasted by Huang H, Shi et al. [11]. It was stated that the Friis’ model does not account for phase margin, since the pulsing loss and polarization shift losses were not taken into account by the Fresnel model as a near field. A simple new model has been suggested that identifies four categories of fading channels from the field dynamics of the amplifier. In comparison to the Friis and Fresnel models, the proposed hybrid model had good performance with field experimental results. For the conceptual scheme in the radio frequency area, the coefficients are based on soil types. Zungeru et al. [12] suggested a pulse power dependent on magnetic induction. Their analytical findings of the Magnetic Modulation derived the pulse strength with a usual electromagnetic field communication channel, an increase in signal-to-noise ratio, and fading channels of variance in node. Shigeru Teruhi et al. [13] implemented a device that incorporates drive-by information congregation and fixed information gathering in order to successfully gather audible detection information. Tests calculating the radio transmission intensity through underground sensor nodes have been implemented in different sub-surface settings to assess the proposed underground radio propagation model. 

WUSNs are made up of sensors that are hidden in the substrate and interact through it with the underlying climate, such as moisture content and density, on the operational controls of WUSNs. The underground sensor nodes constantly need to sense due to precipitation and weather extremes, making remote contact far more difficult than in traditional over-the-air sensor networks. Zhao et al. [14] suggested using sensors to detect strategies to achieve accurate and resource-efficient data collection in complex WUSNs to reduce the path loss through sensory information transfer, energy constraints, and device traffic shaping. They also examined the impact of underground conditions on wireless communications, route possibility, power production, and data aggregation functions in terms of prompting questions about security and availability. The research of an intelligent Wireless Sensor Networks (WSN) for backflow prevention and scale estimating in piping systems was proposed by Sidra Rashid et al. [15]. In their work, autonomous functions such as slow or small leakages in gas and oil pipelines using wireless connectivity and DL are achieved. A sequence of experiments for a site-implemented test platform was used to evaluate the efficiency and strengths in the detection of defects and size approximation in reservoirs. S. Wang and Y. Shin [16] suggested an effective routing protocol using machine learning and Q-learning to analyze strategic planning in structured channels. The authors also extracted the upgrading function of the scheduling scheme by defining the individual hopping incentive metric of duration and energy. Furthermore, they developed a legislative factor to change the proportions between energy conservation and low delay, allowing them to satisfy a variety of needs. The experiments demonstrated that the proposed method provides a better communication range and lower transients. K. I. Wang et al. [17] proposed the WUSNs architecture of agriculture by experimenting with a soil channel model to allow precise simulations in real-world deployments using Long Range Wide Area Network (LoRaWAN) technology.

Silva et al. [18] explored the connection strength properties of the triple messaging services accessible in WUSNs for groundwater pipeline monitoring. The three messaging services in WUSNs, such as received signal frequency, link quality measure, and queue length ratio, were measured. Their analysis indicated that the underground medium is strongly perpendicular and cognitively consistent. An experiment involving many machine learning-based electronic networks was developed and tested in an extracorporeal circuit by X. Tan et al. [19]. The experiments were carried out in a controlled earthquake zone with variables such as vegetative cover and salt content. They presented the principles and instructions for designing the MI tunnel connectivity test platform, which is tremendously hard and sustained owing to the latest communication protocols and wireless transmissions. E. P. Stuntebeck et al. [20] investigated system communication arrangements to determine the structures of multiple distant complex systems. For reference purposes, data from the various sensors are controlled by a centralized terminal through a cellular connection with fewer communication channels available. The sensors must be arranged properly for efficient estimation at the gateway. They also devised an integrated Markov chain method to deal with the task scheduling.

A systematic analysis was presented by Singh et al. [21] to capture the emergence of construction methods in sensor network applications. The benefits and drawbacks of various epochs in development were examined in their study to identify the potential research topics in the wireless sensor unifying framework. The wide range uses of wireless networks in a future era of information and network access, which have received much interest in recent decades, were explained. They also handled the issue of the efficient implementation of “cluster heads” to determine the output and longevity of any wireless sensor network. Scholars have reported a number of models for deploying SNs in massive open areas by Vikrant Sharma et al. [22]. Their studies also looked at the connection error margin and the transmission range of accurate data signals for a network of underground sensors using a subterranean sensor.

The various existing papers could provide solutions for monitoring dynamic underground environments but with a lack of accuracy in decision making in WUSNs. The inaccuracy has been because of conventional methods being used in the proposals in taking decisions under hostile situations. In addition, due to the limited communication range of WSNs in an underground environment, there must be flexibility in increasing the number of sensor nodes. From the assessment and evaluation of the literature surveys, it is inferred that the underground sensor networks certainly in need of DL methods that are accurate and reliable in terms of decision making.

## 3. Deep Learning vs. Machine Learning (ML) in Underground Wireless Sensor Network Environment

The differences between machine learning and DL in underground situations are given as follows:

- DL needs more unlabeled training data than ML does to make accurate decisions, because ML users use less data [23].
- DL is in need of high-performance hardware.
- ML needs certain functionalities to be precisely found by users, but DL creates fresh functionalities by itself.
- ML breaks up the work into smaller pieces before putting the final results together to make a decision. On the other hand, DL fixes mistakes in the order in which they were made [24].
- When compared with ML, DL requires a long time to provide training.
- DL is able to offer sufficient steps and procedures for making decisions.

DL models are more flexible than ANN models, making them more sensitive to overfitting. The number of variables and hidden networks in the network can be reduced, and the network can be pruned after training [25]. Hence, the advantages of DL can be taken for better decision making in monitoring and surveillance applications in underground environments using WSN. This section can be divided into subheadings in order to explain the interpretation and conclusions in a clear and concise way [26].

## 4. Proposed Deep Learning Based Cooperative Communication Channel Model for WUSNs

**Figure 1:**
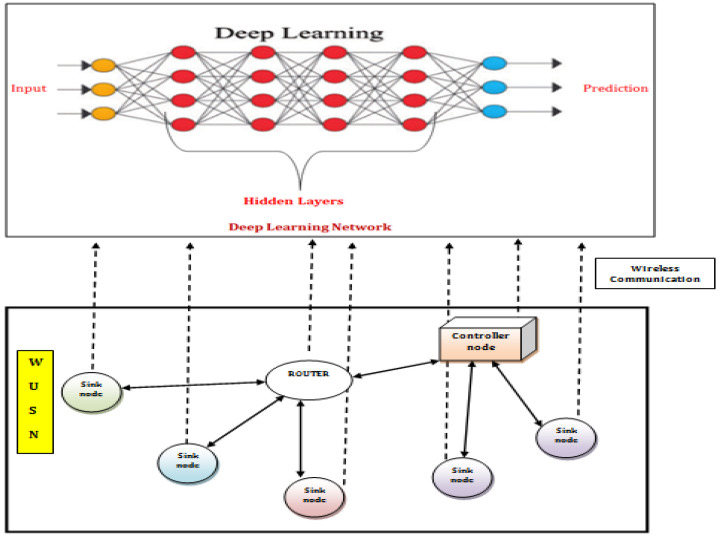
Deep learning network structure in WUSNs middleware.

Figure 1 depicts a proposed DL-based wireless underground sensor network infrastructure with Cooperative communication nodes. The source and sink nodes of cooperative modules have been critical components in monitoring outer communications. The component of a Cooperative communication framework to allow the WUSN and DL networks for efficient communication has been separated. Through the WUSN middleware console, various system components collaborate for computation and storage functions [27]. As a result, the virtualized distributed DL network structure is designed using the WUSN middleware management platform. In the WUSN, nodes with storage capabilities, such as source nodes, sink endpoints, and Cooperative access points, can contribute to building the DL network of WUSN Middleware. The improved process will be communicated to solve the problem of the sink node. Furthermore, the cooperating connections will be the main computational components. Moreover, the Cooperative communication node is embedded in the WUSN middleware controller.

The Deep Learning Auto Encoder (DLAE) is built on top of a distributed DL network. The primary controller has hidden layers and output units. DLAE is responsible for estimating the underground dynamic condition through information in the approach [28]. Local storage package query information needs to be gathered and sent to the server by the sink nodes [29]. The previous information will also be examined in the input layers by the analytics system during the test process. The analytics function summarizes the existing data from many timestamp requests for the input layers in the preprocessing phase. The DLAE then makes a forecast for the known data packet [30]. Depending on the forecast, the WUSN middleware develops a storage approach. Additionally, the storage method is matched up with these storage servers so that preemptive storage can happen over time [31].

In this work, we identify the effects of multiple factors as well as utilize DL tools in evaluating an optimal transmission strategy to decrease transmission loss and intelligent consumption of sensor power [27]. Using the DL method, the exploration of environmental conditions impacts on wireless connectivity in underground surroundings, such as transmission pathway loss, energy consumption, and system bandwidth balancing [32]. As a result, the development of a dependable and powerful data collection transmission is structured as a Cooperative and multi-constrained communication-based limitation.

The intended solution is identified from the surroundings and articulated together for numerous limitations using DL approaches. An adaptive threshold strategy has also been established to benefit from diverse networks [33]. The proposed methodology aims to increase efficiency by means of transmission loss and energy constraints. Additionally, the proposed method can enhance transmission dependability and reduce network costs.

### 4.1. Energy Model for DL Based Cooperative WUSN Communication Channel

One of the most common WUSN routing algorithms is Cooperative communication concentrated routing protocols, in which sensors that make up the network are partitioned into Cooperative cluster nodes subject to constraints. For each of these groups, they work together as a large configuration to collect sensed data [34]. The standard method to figure out how much energy has been lost because transmitters need power to send a (l) level of compliance over a (w) wavelength is:

E_T_ (l,w) = l (E_tx_ + E_sf_ × r^2^), whether h < h_0_ (1)

E_T_ (l,w) = l (E_tx_ + E_pm_ × r^4^),whether h ≥ h_0_ (2)

In order to attain a (l) level of functionality, the receiving nodes will also burn a significant amount of energy and the wasted energy, by the receiver:

E_R_ (l) = l × E_rx_ (3)

(E_tx_) and (E_rx_), respectively, reflect the actual capacity of each transmission used to power the broadcasters and receiver radio equipment. (E_sf_) and (E_pm_) reflect the power used by packages broadcast to operate the radio amplifier in attenuation and modulating methods over several paths. The below link between (E_sf_) and (E_pm_) can also be used to compute the threshold width [35].

(4) $$h_{0}=\sqrt{E_{\mathrm{sf}}+E_{\mathrm{pm}}}$$

The total power consumption is compensated by the connection between other endpoints in a communication network. The primary function of communications is not only to find the shortest path from a source to a receiver but also to find a far more efficient method to extend the process duration, which could be accomplished using DL methods [36]. Following the detection of environmental elements, the data will be transferred to the source [37]. Information exchange energy consumption (E_tx_) among sensing devices can be expressed as

E_tx_ = E_dtx_ × q + £_ampl_ × lw^ar^ (5)

where,

- q denotes the maximum number of information packets transmitted;
- ar denotes the level of aspect ratio depending on the wireless communication case;
- lw is the length and width of the sensor nodes, which is denoted by d.
- £_ampl_ is the set of enhancing equations needed to achieve a low bit error rate and reliable broadcaster responses.
- E_dtx_ is the energy dissolute to function for the sender/receiver and is expressed by

E_dtx_ = V_cc_ × C_tp_/q_dr_ (6)

where

- V_cc_ indicates the operational power.
- C_tp_ species the power for communication procedure
- q_dr_ represents the information transmit velocity

The energy consumed for data receiving can be expressed as

E_Rx_ = E_drx_ × q (7)

### 4.2. DL Based Routing Model in WUSN

**Figure 2:**
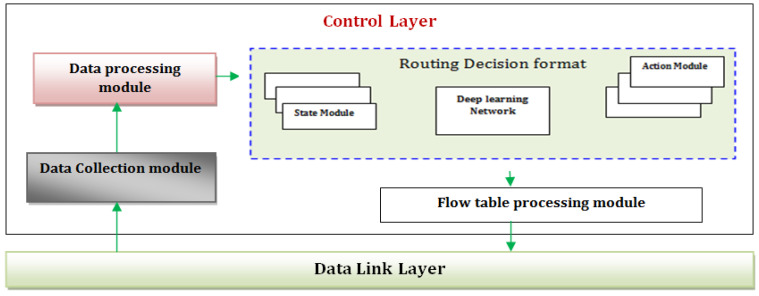
DL based Routing model.

DL can maximize the utility by determining the optimum route through ongoing testing with the environment. DL combines a hierarchical machine learning model with reinforcement systems [38]. It is necessary to train variables using the current values of reinforcement learning and to replace the reinforcement learning’s Q value table with a neural network [39]. Depending on the type of Cooperative communication channel construction and the benefits of DL in terms of evaluation and decision, the proposed route planning strategy is based on labeled data to meet the Q-value table of DL. The DL-based Routing model is depicted in Figure 2. The control layer mainly includes data collection module, data processing module, routing decision module and processing module. Data collection and processing are done before, it is given to the routing decision format in order to get undergo flow table processing module. Here, the deep learning actually refers to the decision making process which following the routing decision format.

The essential aspects of the central controller in the overall system design are information collection domain, information analysis, routing choice, and route table information processing. The information collection domain collects a piece of data for data transfer to reduce the dimensionality before route table information processing in the traffic method. Finally, the path discovery decision module includes a traffic method-based method [40,41]. A DL neural network is used in the routing selection module to acquire sufficient knowledge from the information collection domain and receive environmental monitoring attributes using DL neural networks [29].

The trained neural network uses classification methods to accomplish the decision making task. It may turn the current circumstances into tasks using suitable procedures and evaluate various moving platforms [42]. Finally, the Q-value tables created by supervised learning can be linked into key entities to act as the foundation for routing decisions. In this work, a multi-Cooperative communication channel path routing scheme using deep r training is built successfully:

Multi-Cooperative communication channel path routing Algorithm 1 based on Deep Learning:
**Algorithm 1** Multi-Cooperative Communication Channel Path RoutingInitiate position = IP, Final position = fp, Quality of Service intensity = δ Least obtainable bandwidth of present connection = bw available the volume amount of traffic = bw_min_ (s, r) routing = routing values of sender/receiver pp ← present topology While bw_min_ > 0 Configure monitoring valueset preprocessing condition of packets = Sp, Initialize buffer pool storage amount Loop traversal:   C = the data package’s choice of operation,   Perform the operation C to get r_e_, s_e_   Keep these values r_e_, s_e_, C to buffer pool storage   If the information in the memory pool is sufficient   Calculate _ Computation of the net carry away percentage Computation Q (r_e_, s_e_, C; θ)   From Eval _ Net to target _ Net, there are N phases.   Target _ Subtract the sampling computation from the net value Computation Q (r’_e_, s’_e_, C’; θ’) Preparation neural network to obtain direction Break Else   if ip’ = fp Else   ip = ip’   Final position Routing (s,d) ← Routing Using two nodes in this routing, least amount obtainable bandwidth If bw available ≤ bw_min_ t[nodes] = 0, to update t

The packet loss of traffic frequency is defined as the ratio of the total amount of bandwidth lost by all traffic to the total amount of bandwidth requested by all traffic.

Loss rate = ∑_iBw loss(x)/∑_iBw (x)(8)

Bw shows the bandwidth of the xth traffic. xth traffic is the formula for specifying traffic bandwidth loss.

Bw^x^_loss_ = Bw_x min_BwR^x^_bw_ (9)

R^x^_bw_ includes the overall link throughput of the xth traffic’s forwarded pathway; _min_BwR^x^_bw_ is the route’s minimal connection frequency. 

## 5. DL Based Transmission Path Selection in WUSN

The ambient and contextual ubiquitous computing elements in implementing strategic and energy-efficient sensory data collection broadcasts are carried out in underground wireless environments. The operating segments of sensors have been used to save energy and limit the number of packets with errors [37]. Sensor information is sent to sensor nodes through a process called a Cooperative communication transmission channel. The creation of an adaptive threshold strategy is also executed that learns from its interaction with the environment in order to collect the data in order to make an effective contribution [35].

Fp = (wusn_sn_, W_way_, C_way_, Poss_way_, Eng_way_, Ebl_way_) (10)

where

- wusn_sn_ is the information from the original sensor network being sent to sensor node
- W_way_ is a set of sensor nodes that participate in the transmission of sensory input from the WUSN to the sensor node.
- C_way_ is a series of connections between two points of W_way_.
- Poss_way_ is a collection of possibilities for different paths of Epath.
- Eng_way_ is a collection of energy necessities for the transmission of sensory information from WUSN_sn_ to SN.
- Poss_way_ is a collection of possibilities for different paths of wusn_x_ to wusn_x+1_

Poss_way_ = {(Poss_way_ (wusn_sn_, wusn) Poss_way_ (wusn_sn_, wusn_x+1_) Poss_way_ (wusn_way_, SN)} (11)

Eng_way_ is a collection of energy necessities for the transmission of sensory information from wusn_sn_to SN.

Eng_way_ = {Eng_way_ (wusn_sn_, wusn) Eng_way_ (wusn_sn_, wusn_x+1_) Eng_way_ (wusn_way_, SN)} (12)

Ebl_way_ is a set of variables for balancing load of Eng_way_.

Ebl_way_ = {Ebl_way_ (wusn_sn_, wusn) Ebl_way_ (wusn_sn_, wusn_x+1_) Ebl_way_ (wusn_way_, SN)} (13)

The gathered information is regularly forwarded to SN via fp under the constraints of Poss_way_, Eng_way_, and Ebl_way_ to obtain reliability and energy efficiency, with the absolute maximum minimum cost method.

wusn_x.energy_ > engcst (wusn_x_) £ fp × V_path_ (14)

fpPoss_way_ (wusn_x_, wusn_x+1_) ≥ fp × W_way_ (15)

wusn_x_*,* wusn _x+1_ £ fp × W_way_ (16)

fpC_way_ (wusn_x_, wusn_x+1_) = 1 (17)

avg (fpPoss_way_) ≥ thsd_ap_ (18)

## 6. Experimental Results and Discussion

The design and evaluation of the proposed WUSN have been carried out with the Qualnet Simulator. Methods such as ECCAR, EHOR, non-cooperative, and DER are used to evaluate the performance of the WUSN environment. A group of 25 sensor nodes in a WUSN is scattered in an area covering 100 m × 100 m. It is also assumed that a pair of sensor nodes can communicate between them if the radius is 25 m.

- The maximum path loss can be computed using the highest transmission rate and the shortest reception strength measurements [43]. It is understood that the path loss is affected by the distance between the source and the destination. The operating frequency is set at 900 MHz.
- Based on the energy equation to fix the energy of the underground sensors to 100 J and the packet size to 125 bits [44].
- The ratios for power consumption and the dynamic routing factor are chosen to highlight the equal priority of electricity usage and load balancing factor. The path probability is chosen to ensure the reliability of the transmission pathways [27].

The proposed system was evaluated with a specific number of cooperative nodes in Cooperative communication using a DL-based routing model and the quantity of energy consumed was also compared.

**Figure 3:**
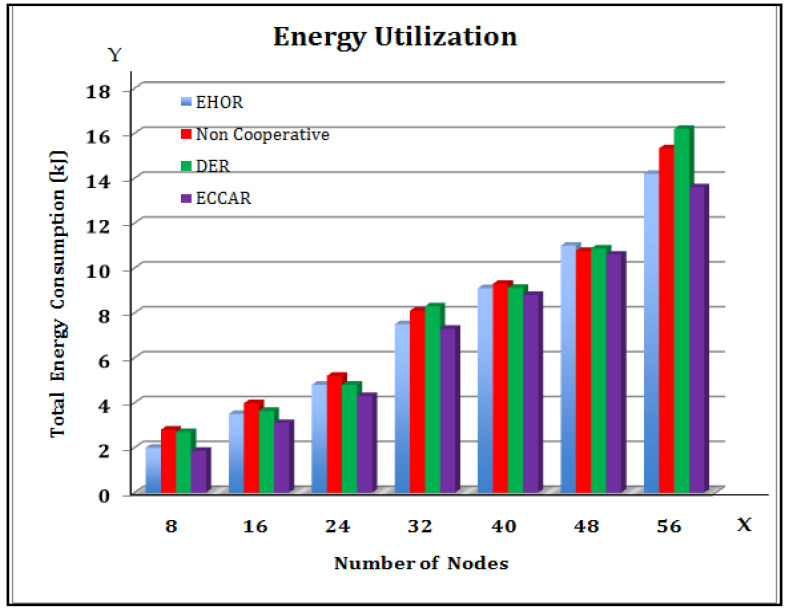
Overall Energy Utilization.

Figure 3 presents a comparison of EHOR non-Cooperative routing and Dynamic DER methods. In Figure 3, the X axis represents the number of nodes, and the Y axis represents the Total Energy Consumption (kJ). It is suggested that the ECCAR is effectively used for various numbers of Cooperative nodes to calculate the overall energy utilization. It has also been seen that as the number of Cooperative nodes grows, so does the energy usage of the four routing algorithms. If the number of cooperating nodes increases, then the number of qualifying forwarding nodes impacts the increased energy utilization for transmitting, receiving, and sometimes even sleeping modes. The suggested ECCAR routing technique improves previous EHOR, non-Cooperative routing, and DER routing mechanisms. Thus, it is more suitable to find a network that works together. Routing problems use less energy.

**Figure 4:**
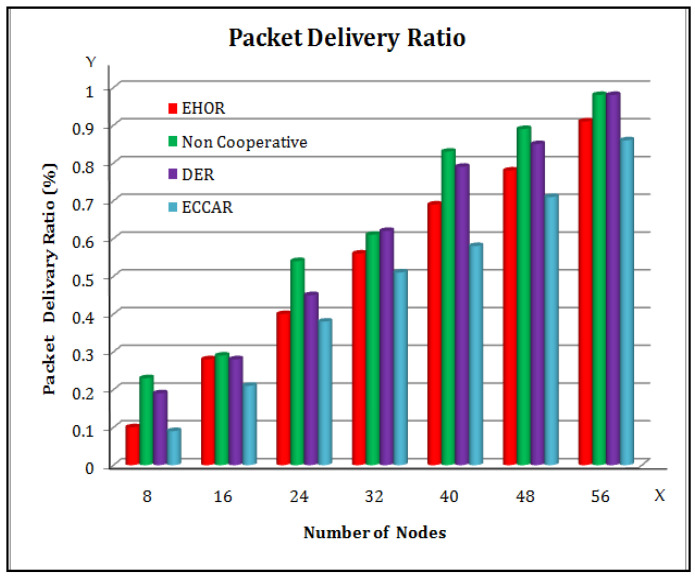
Packet Delivery Ratio.

The effectiveness of the four different methods in terms of packet delivery ratio of Cooperative communication nodes is analyzed in Figure 4. In this Figure 4, X axis represents the number of nodes and Y axis represents the Packet Delivery Ratio (%). It has been studied that as the intensity increases, the packet delivery ratio starts improving proportionally [45]. This is due to the fact that while Cooperative communication network nodes rise, additional networks have the possibility of being identified as acceptable Cooperative nodes, resulting in a greater packet delivery ratio. When the scalability increases, EHOR, DER, and non-cooperative routing methods can attain higher packet delivery ratios. The ECCAR packet delivery ratio is heavily influenced by the subsurface channeling diameter and the position accuracy underground of the wireless sensor platform. In addition to that, the number of sensor nodes in the underground is affected by the passive movement of cooperative nodes in the WUSN, thus reducing the ECCAR packet delivery ratio. Hence, in the channel selection process, the detecting and recovering modes are employed to increase the packet delivery ratio in ECCAR to produce great results in the WSU networks.

**Figure 5:**
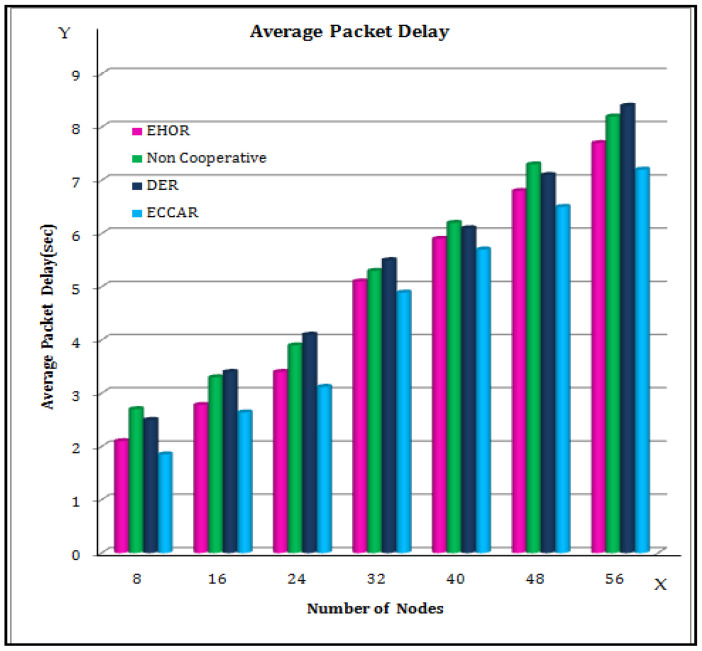
Average packet Delay.

The influence of node density on the average packet delay of the methods is depicted in Figure 5. In Figure 5, the X axis represents the number of nodes, and the Y axis represents the Average packet delay (s). The average packet delay of the techniques decreases as the number of sensor nodes increases, due to the ability to discover more qualifying nodes in their region to relay packets [46]. It is also found that the ECCAR has a longer average packet delay than the other methods. Hence, the packets in ECCAR are only transported within the route underground constructed from transmitter to receiver. On the other hand, the underground nodes may not be as close to the upper sink as the surface nodes, which make the average packet delay longer.

**Figure 6:**
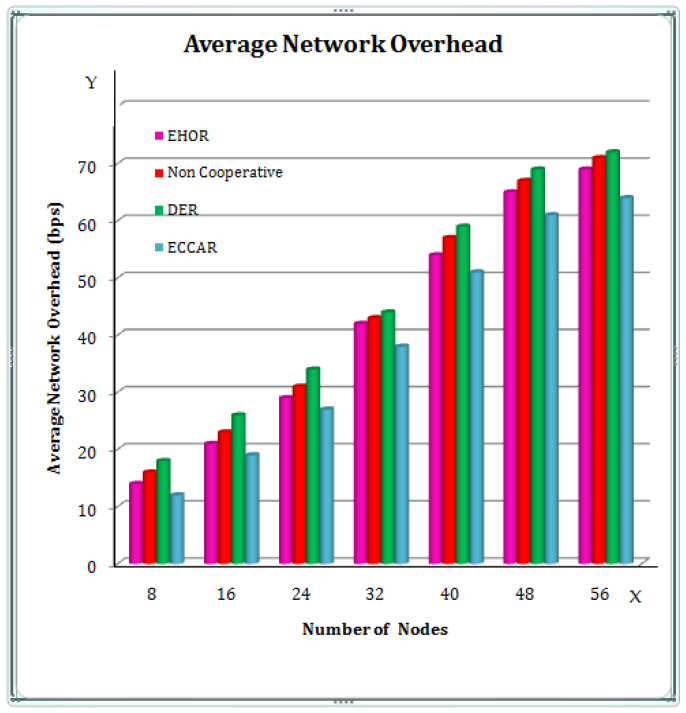
Average Network overhead.

Figure 6 displays the effects on the average network overhead of the four techniques at various node concentrations. In Figure 6, the X axis represents the number of nodes, and the Y axis represents the Average Network overhead (bps) [47,48]. When compared to other approaches, it can be seen that ECCAR has a lower average network overhead. The reason for this is that in ECCAR, a large number of Cooperative nodes collaborate on the packet transmission procedure using an efficient duplicate packet attenuation approach. However, for the reason that it can discover a solution, the average overhead is lower than with other approaches. Therefore, the suggested method has less average network overhead than the other methods.

**Figure 7:**
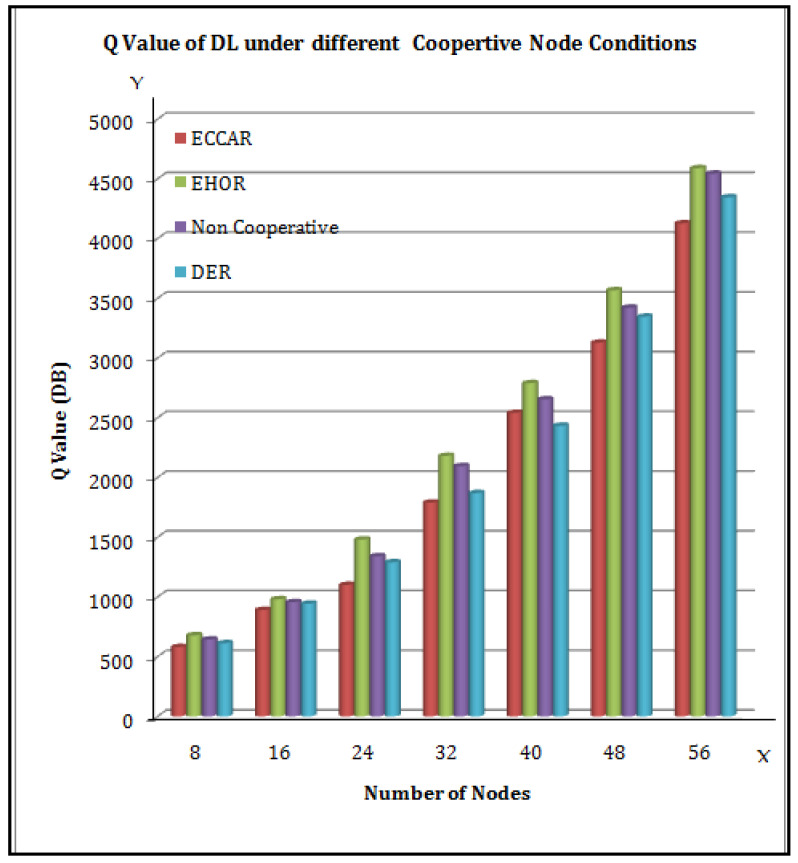
Q Value of DL under different Cooperative node conditions.

Figure 7 illustrates the Q value of DL for various Cooperative nodes, as well as various routing strategies. In Figure 7, the X axis represents the number of nodes, and the Y axis represents the Q Value (DB). Investigations have been conducted to compare EHOR, DER, and ECCAR with different quantities of Cooperative nodes on a platform with DL-Q value. It was found that a Cooperative communication node with the lowest Q value helps the system to choose the cognitive node with the lowest power level.

**Figure 8:**
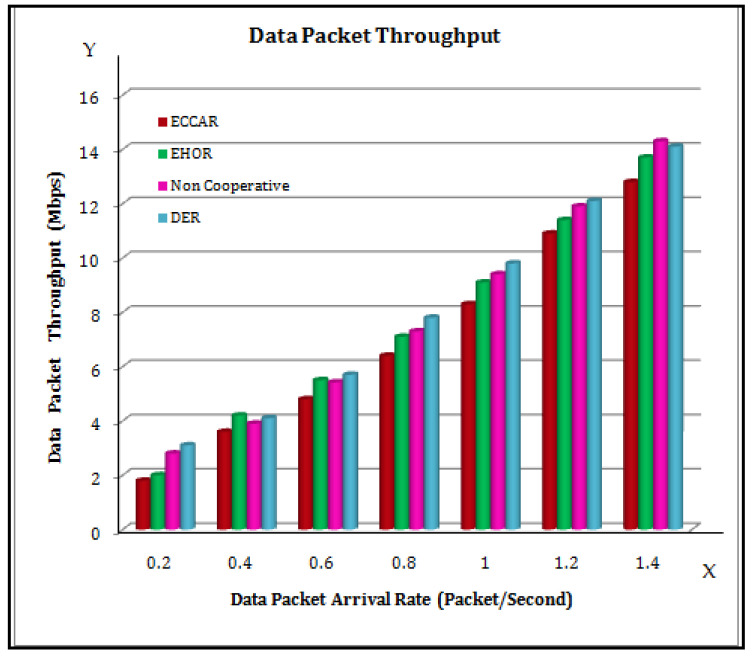
Data Packet Throughput.

Figure 8 illustrates the data packet arrival rate, which is directly proportional to the throughput. In Figure 8, the X axis represents the data packet arrival rate (packet/s) and the Y axis represents the data packet throughput (Mbps). As a result, ECCAR has some superiority over other methods in achieving better throughput optimization.

**Figure 9:**
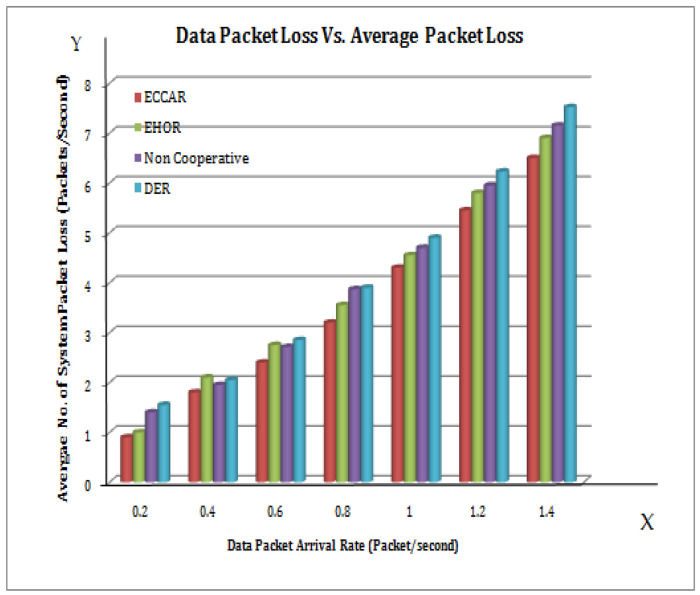
Data packet loss vs. Average packet loss.

From Figure 9, it is inferred that as the data packet arrival demand rises, the network’s average number of packet losses tends to increase as well. In Figure 9, the X axis represents the data packet arrival rate (packet/sec) and the Y axis represents the average number of system packet losses (packet/sec). The number of bytes delivered by the network per unit time grows as the arrival rate of the data packet increases [25,49]. Figure 3, Figure 4, Figure 5, Figure 6, Figure 7, Figure 8 and Figure 9 have been compared individually between them and it is inferred that since the network is prone to failure, the higher the quantity of data messages have been delivered with the greater the percentage of packets lost. Hence, it is concluded that the efficiency of ECCAR has the lowest average number of packet network instability.

## 7. Summary of Contribution

As DL is a group of ML algorithms that provide a model with high-level substitutions in data with architectures comprising more nonlinear changes, the proposed DL Cooperative communication model relies on artificial neural networks to ensure the best decision making at appropriate times. The DL-based model proposed has firmly undergone proper training to enhance the reliability of the training processes to handle larger amounts of data efficiently. In the first phase of training, the Deep ML process begins by labeling huge quantities of data before finding out their functionalities. The proposed model also permits the use of more difficult groups of features as it is capable of producing convenient solutions with layers of neurons. Because the DL-based Cooperative communication model is highly intelligent, the WUSN could be used for surveillance in any hostile environment.

## 8. Conclusions

A DL-based Cooperative communication channel model for WUSNs has been presented. The use of DL has been proven to be effective in terms of accuracy when trained with an enormous quantity of information to construct smart assessments in underground wireless environments. The proposed ECCAR, EHOR, non-Cooperative, and DER methods have been used to investigate the performance of WUSN to evaluate the QoS parameters such as transmission time, throughput, and packet loss. From the results, it is understandable that DL has been working well with the larger amounts of data on hand in terms of scalability. Its efficiency also increases as the datasets increase. Furthermore, comparison of the proposed model with the existing work is also made. From the simulation, it is also inferred that the unnecessary energy consumption by individual nodes is reduced as a result of the proper utilization of the Cooperative sensor nodes. The proposed Deep Learning-based Cooperative communication channel model for WUSNs is unique as it manages data inputs constantly to determine transparent decisions in the sharing of resources. Future work will focus on issues such as the Min-Max problem of the required Quality of Service (QoS) metrics, estimation of multi-hop routes using mobile relays, and the development of deterministic channel state models using DL-based WUSN.

## Data Availability

Not applicable.
